# A Case-Control Study on Factors Associated With Secondary Amenorrhea Among the Medical Students of Universiti Malaysia Sabah

**DOI:** 10.7759/cureus.47625

**Published:** 2023-10-25

**Authors:** Win Win Than, M Tanveer Hossain Parash, Nathira Binti Abdul Majeed, Khin Nyein Yin, Dg Marshitah Binti Pg Baharuddin, Ehab Helmy Abdel Malek Fahmy, Mohd Nazri Bin Mohd Daud

**Affiliations:** 1 Obstetrics and Gynecology, Faculty of Medicine, Asian Institute of Medicine, Science and Technology (AIMST) University, Bedong, MYS; 2 Biomedical Sciences, Faculty of Medicine and Health Sciences, Universiti Malaysia Sabah, Kota Kinabalu, MYS; 3 Obstetrics and Gynecology, Hospital Wanita Dan Kanak-Kanak Sabah, Kota Kinabalu, MYS; 4 Rehabilitation Medicine, Hospital Universiti Malaysia, Kota Kinabalu, MYS; 5 Rehabilitation Medicine, Faculty of Medicine and Health Sciences, Universiti Malaysia Sabah, Kota Kinabalu, MYS; 6 Obstetrics and Gynecology, Faculty of Medicine and Health Sciences, Universiti Malaysia Sabah, Kota Kinabalu, MYS; 7 Public Health Medicine, Faculty of Medicine and Health Sciences, Universiti Malaysia Sabah, Kota Kinabalu, MYS

**Keywords:** follicle-stimulating hormone, case-control studies, luteinizing hormone, body mass index, amenorrhea

## Abstract

Background and aim: This study aimed to evaluate the association between body mass index (BMI), anxiety, stress, depression, hormones, and secondary amenorrhea among female medical students at Universiti Malaysia Sabah (UMS).

Methods: In this case-control study, UMS undergraduate female medical students aged 19-25 years who did not menstruate in the last three months (with a previous history of a regular menstrual cycle) or six months (with a history of irregular menstruation) were included as cases (40 students), and students with similar criteria but no menstrual irregularities were recruited in the study as controls (40 students). The study was conducted at Polyclinic UMS from January 1, 2021, until December 31, 2022. The chi-squared test and odd ratio examined the association of the above-mentioned factors with the secondary amenorrhea. A p-value less than 0.05 was considered significant, and an odds ratio if the confidence interval did not contain one was considered significant.

Result: Both the groups had a similar frequency of different BMI grades. The cases exhibited significantly higher levels of depression, anxiety, and stress than the controls. Again, the cases demonstrated higher estradiol (E2), testosterone, and thyroid-stimulating hormone (TSH) levels and lower levels of luteinizing hormone (LH) than those with regular menstruation. The research also revealed that a one-unit decrease in follicle-stimulating hormone (FSH) levels corresponds to a threefold increase in the risk of experiencing secondary amenorrhea, while the risk escalates to fourfold for LH. Moreover, E2, testosterone, and TSH levels exhibited protective effects on secondary amenorrhea.

Conclusion: Anxiety, serum LH, and FSH were significantly associated with secondary amenorrhea. Future studies should address the diurnal variation of the hormones and consider the participants' circumstances to get a proper effect of hormonal influence and stress.

## Introduction

Menstruation is considered an indicator of women's health, and it is a unique phenomenon that represents the beginning and end of reproductive age. Understanding menstruation patterns and factors affecting it may improve understanding of menstrual characteristics, proper management, and menstrual issues [1]. A regularly (every 21-45 days) menstruating woman is considered to have secondary amenorrhea if she has not menstruated in three months. For an irregularly menstruating woman, amenorrhea should last six months to diagnose it as secondary amenorrhea. Hypothalamic amenorrhea (HA) and polycystic ovary syndrome (PCOS) are the two most common causes of secondary amenorrhea other than pregnancy [2].

Hypothalamic amenorrhea (HA) caused by deficient secretion of hypothalamic gonadotropin-releasing hormone (GnRH) accounts for approximately 30% of cases of secondary amenorrhea in women of reproductive age. It leads to failure of pituitary gonadotropins and gonadal steroid release. Functional hypothalamic amenorrhea (FHA) is defined as hypothalamic amenorrhea (HA) occurring in the absence of a structural lesion and is predominantly caused by significant weight loss, intense exercise, or stress [3]. FHA is evidenced by low serum estradiol and gonadotropins, which lead to increased bone turnover and rapid bone loss [4].

Higher than normal levels of gonadotropins in females also lead to reproductive pathologies, most notably, polycystic ovary syndrome (PCOS), characterized by an increase specifically in luteinizing hormone (LH) and leading to elevated LH/follicle-stimulating hormone (FSH) ratio [5]. PCOS is a prevalent endocrinopathy in females, characterized by chronic oligo-anovulation, hyperandrogenism, and polycystic ovaries, all of which can worsen the quality of life for these patients [6]. The changes in female hormone levels are associated with health behaviors, obesity, and stress. Research has revealed that elevated stress levels can impact the hypothalamic-pituitary-adrenal (HPA) axis activity. Conversely, a high body mass index (BMI) has been observed to have an impact on sex hormone-binding globulin (SHBG), the free androgen index (FAI), testosterone, and insulin levels. Thus, previous studies have reported a significant association between lifestyle and menstruation [1].

Several researchers have found medical students to have higher stress, anxiety, and depressive symptoms due to the challenging and demanding nature of the medical school curriculum [7-14]. Owing to the curricular commitment, the scope of physical activities is limited [15]. Hence, medical students are prone to be exposed to obesity [16]. The prevalence of overweight and obesity among medical students at Northern Border University, Arar, Saudi Arabia, was 21.7% and 8.4%, respectively, in a study by Mehmood et al. [17]. In their reflections on the Peninsular Malaysian population, Gopalakrishnan et al. and Boo et al. found that 30.1% of the students were overweight or obese [18,19].

Again, stress has the potential to significantly contribute to or even trigger menstrual irregularities. Evidence indicates a connection between stress and menstrual irregularities [20,21]. Additionally, a notable prevalence of menstrual issues has been noticed among students pursuing studies in medicine and health sciences [22-25]. In their research, Sood et al. and Clarvit found no association between perceived stress and menstrual problems [15,26]. In contrast, other researchers reported that stress scores could predict irregular menstrual cycles [27-32]. Hence, the present study investigated the association of BMI, anxiety, stress, depression, and hormones with secondary amenorrhea among female medical students from Universiti Malaysia Sabah (UMS).

An earlier version of this article was previously posted to the Research Square preprint server on February 10, 2023.

## Materials and methods

This case-control study was carried out during the period starting from January 1, 2021, until December 31, 2022.

Inclusion and exclusion criteria

UMS undergraduate female medical students with an age range of 19-25 years who attended the Polyclinic UMS during the study period were included in the study as cases if they did not menstruate in the last (1) three months, with a previous history of a regular menstrual cycle or (2) six months, with a history of irregular menstruation. Female students with similar criteria but no menstrual irregularities were recruited in the study as control.

The students who received hormonal treatment in the past six months, with organic lesions of the genital tract, bleeding disorders, and pregnancy, were excluded from the study.

Sampling

The minimum sample size required for this study was 80 (where two-sided confidence level=95%, power=90%, the ratio of controls to cases=1, percent of controls exposed: 25%, odds ratio=5, and percent of cases with exposer=62.5%) [33]. A total of 80 participants who fulfilled the inclusion criteria were included through stratified random sampling.

Study procedure

After getting written informed consent, all respondents were asked to complete filling out self-administered questionnaires for age, level of education, and menstrual histories, such as their age at menarche, regularity of menstrual cycle, most prolonged duration without the period, usage of hormonal medication to regulate their menstruation in previous six months, any intermenstrual bleeding and its time, past medical history, current weight gained or lost, any engagement in exercise and sports, and any history of having bone density scan. Of these respondents who fulfilled the selection criteria, 40 subjects were randomly recruited as cases, and the other 40 were recruited as controls. Then, the respondents' depression, anxiety, and stress were measured by Depression, Anxiety, and Stress Scale-21 (DASS-21), and the respondent's height and weight were measured to calculate BMI, and blood samples were collected for hormonal studies. Blood samples were analyzed the same day, and the results were available within 24 hours. Hormone levels of follicle-stimulating hormone (FSH), luteinizing hormone (LH), estradiol (E2), total testosterone (TT), prolactin (PRL), thyroid-stimulating hormone (TSH), and free thyroxine (T4) were measured by enzyme-linked immunosorbent assay (ELISA) method using Cobas e411 analyzer (Basel, Switzerland: Roche Holding AG).

Ethical permission

The research underwent a thorough review by the Medical Research Ethics Committee of the Faculty of Medicine and Health Sciences, Universiti Malaysia Sabah, and the ethical approval code for this research is JKEtika 1/20 (12). The study complied with the ethical principles outlined in the appropriate edition of the Declaration of Helsinki, initially established in 1975 and revised in 2000.

Data analysis

At first, the data were screened for normality and were not normally distributed. Hence, median, quartiles, and interquartile range (IQR) were used to describe the data. Mann-Whitney U tests were performed to compare the variables between the two categories, and the odds ratio determined the association between the variables. A p-value less than 0.05 was considered as the level of significance. All the statistical analysis was performed using SPSS software version 27.0 (Armonk, NY: IBM Corp.).

## Results

Within the group of participants, the median age for individuals experiencing regular menstruation was 23 years, with an interquartile range (IQR) of 2. In contrast, those with irregular menstruation had a median age of 22 years and an IQR of 2.5. Additionally, both groups exhibited a median age of menarche at 12 years, with IQRs of 1 and 1.5, respectively (Table 1).

**Table 1:** Descriptive statistics of the respondents (n=80). IQR: interquartile range

Factors	Menstruation	Minimum	Median	IQR	Maximum
Age (years)	Control	21	23	2	25
	Case	19	22	2.5	27
Menarche (years)	Control	10	12	1	15
	Case	9	12	1.5	18

**Figure 1:**
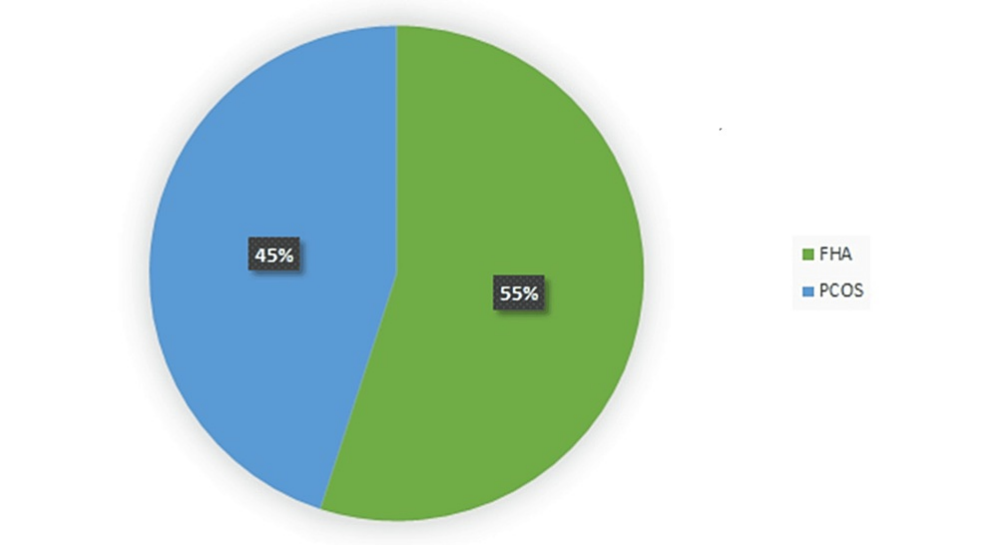
Frequency of types of secondary amenorrhea among the respondents (n=40). FHA: functional hypothalamic amenorrhea; PCOS: polycystic ovary syndrome

Among the cases, the majority (22 persons) of the participants had FHA, and the rest of the participants (18 persons) were diagnosed with PCOS (Figure 1). They were diagnosed based on their clinical evidence.

**Figure 2:**
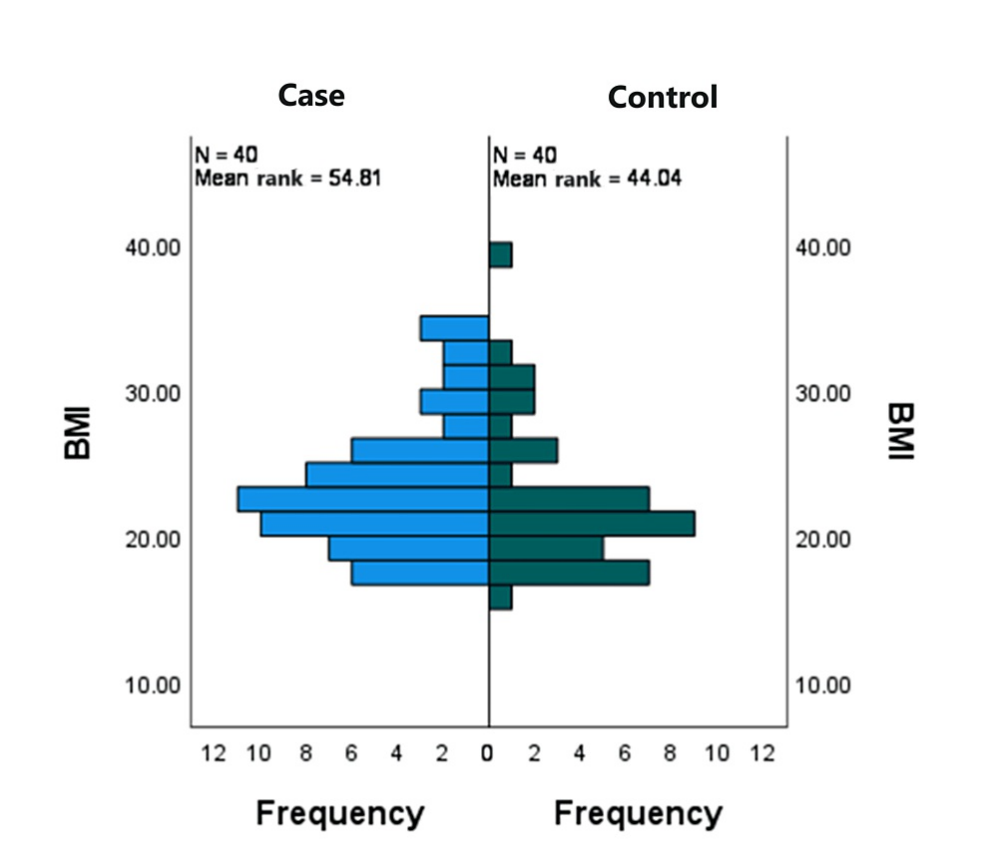
Comparison of BMI among the case and control groups (n=80).

As the subjects were selected as matched pairs, both the groups had a similar frequency of different BMI grades (Table 2 and Figure 2). As the data are not normally distributed, but the respondents were randomly selected, and the shape of the data appears to be the same, the Mann-Whitney test was performed to test the following hypothesis: H_0_=there is no difference in BMI between the two groups and H_1_=there is a difference in BMI between the two groups.

**Table 2:** Distribution of DASS-21 score among the respondents (n=80). DASS-21: Depression, Anxiety, and Stress Scale-21

Factors		Control		Case	
		Frequency	Percentage	Frequency	Percentage
Depression	Normal	36	90.0	31	77.5
	Mild	3	7.5	7	17.5
	Moderate	1	2.5	2	5.0
Anxiety	Normal	35	87.5	23	57.5
	Mild	4	10.0	2	5.0
	Moderate	1	2.5	13	32.5
	Severe	0	0	2	5.0
Stress	Normal	39	97.5	36	90.0
	Mild	1	2.5	3	7.5
	Moderate	0	0	1	2.5
	Total	40	100.0	40	100.0

**Figure 3:**
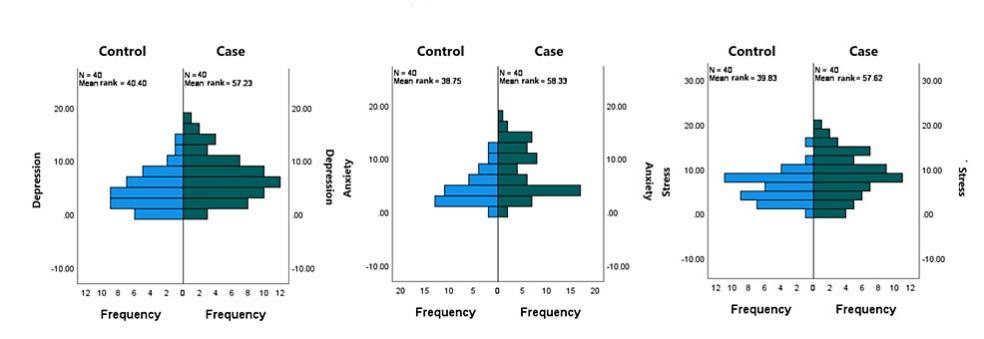
Frequency of DASS-21 score among the case and control groups (n=80). DASS-21: Depression, Anxiety, and Stress Scale-21

The p-value was higher than the significance level, α=0.05 (Table 2). There was insufficient evidence to reject the null hypothesis. There were some apparent differences in the frequencies of depression, anxiety, and Stress (DASS-21 score) between the two groups (Table 3 and Figure 3), which was tested for the following hypothesis: H_0_=the two groups have no difference in the DASS-21 score and H_1_=there is a difference in DASS-21 scores between the two groups.

**Table 3:** Distribution of BMI score among the respondents (n=80).

Factors		Control		Case	
		Frequency	Percentage	Frequency	Percentage
BMI	Underweight	8	20.0	4	10.0
	Normal	22	55.0	24	60.0
	Overweight	6	15.0	7	17.5
	Obese	4	10.0	5	12.5
	Total	40	100.0	40	100.0
Mann-Whitney U test			Standard error	z-Value	p-Value
1458.50			142.11	1.819	0.069 ns

As the samples were independent random samples and the shapes of the distributions were the same, the requirement of the Mann-Whitney test was fulfilled. The p-values for depression (0.004), anxiety (<0.001), and stress (0.003) were less than the level of significance, α=0.05 (Table 4). So, the null hypothesis was rejected. There was sufficient evidence to conclude that the DASS-21 score of the participants with irregular menstruation differed from the matched control group.

**Table 4:** Comparison of DASS-21 between case and control groups (n=80). DASS-21: Depression, Anxiety, and Stress Scale-21

Factors	Mann-Whitney U test	Standard error	z-Value	p-Value
Depression	1604.00	111.53	2.854	0.004
Anxiety	1670.00	141.49	3.322	<0.001
Stress	1627.50	141.65	3.014	0.003

Table 5 demonstrates that the medians of LH, estradiol, testosterone, and TSH differed from the matched control group among hormones that could influence menstrual irregularity.

**Table 5:** Hormonal profile of the respondents (n=80). LH: luteinizing hormone; FSH: follicle-stimulating hormone; TSH: thyroid-stimulating hormone

Factors	LH (mIU/mL)		FSH (mIU/mL)		Estradiol (pmol/L)		Testosterone (nmol/L)		Prolactin (ng/mL)		Free T4 (pmol/L)		TSH (mcIU/mL)	
	Control	Case	Control	Case	Control	Case	Control	Case	Control	Case	Control	Case	Control	Case
Min	3.07	1.56	2.5	1.18	28.59	30.42	0.19	0.36	6.46	3.07	13.95	15.41	0.43	0.47
Q2	7.30	13.74	5.48	6.28	224.13	185.41	0.98	1.44	14.89	17.21	20.19	19.11	1.13	1.45
IQR	4.41	10.29	3.59	2.71	423.47	335.25	0.5	1.82	9.55	21.66	4.94	21.09	0.67	1.95
Max	24.6	83.12	28.34	19.1	1808.12	1085.20	2.27	2.91	34.87	61.72	29.42	28.2	4.25	5.72

**Figure 4:**
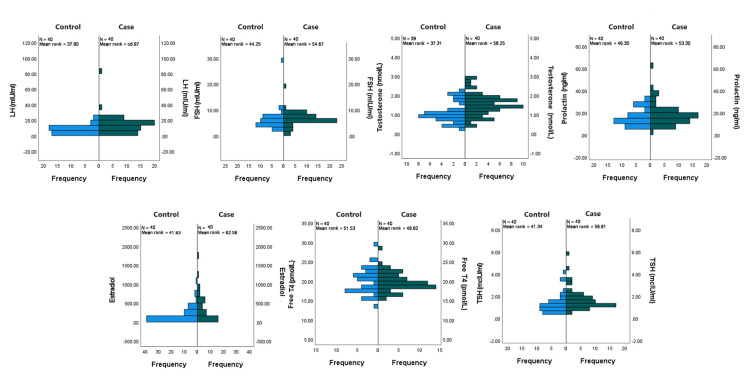
Comparison of hormone profiles among the case and control groups (n=80). LH: luteinizing hormone; FSH: follicle-stimulating hormone; TSH: thyroid-stimulating hormone

The difference in the hormone profile was tested under the following hypothesis: H_0_=there is no difference in hormone profile between the two groups and H_1_=there is a difference in hormone profiles between the two groups. Besides the random distribution, Figure 4 demonstrates similar shapes for hormone profiles for the two groups. So, the assumptions for the Mann-Whitney test were achieved.

The p-values of Table 6 for LH, testosterone, estradiol, and TSH were lower than the significant level α=0.05, but for FSH, prolactin and free T4 were higher than the significant level. There was sufficient evidence to reject the null hypothesis for LH, testosterone, estradiol, and TSH, but the evidence was insufficient to reject the null hypothesis for FSH, prolactin, and free T4. Hence, there was a significant difference in LH, testosterone, estradiol, and TSH levels.

**Table 6:** Comparison of hormone profile among the case and control groups (n=80). LH: luteinizing hormone; FSH: follicle-stimulating hormone; TSH: thyroid-stimulating hormone

Factors	Mann-Whitney U test	Standard error	z-Value	p-Value
LH	1708.00	142.13	3.574	<0.001
FSH	1450.00	142.12	1.759	0.079
Testosterone	1665.00	139.63	3.545	<0.001
Prolactin	1368.00	142.13	1.182	0.237
Estradiol	680.00	138.15	-3.521	<0.001
Free T4	1159.00	142.13	-0.288	0.773
TSH	1566.50	142.12	2.579	0.010

The odds ratio was used to examine the relationship between depression, anxiety, stress, and hormone profiles in relation to secondary amenorrhea (Table 7).

**Table 7:** Association BMI, stress, and hormones with secondary amenorrhea (n=80). LH: luteinizing hormone; FSH: follicle-stimulating hormone; TSH: thyroid-stimulating hormone

Secondary amenorrhea vs other factors (BMI, depression, anxiety, etc.)		Odds ratio	95% confidence interval	
			Lower	Upper
BMI	Normal/obese	1.286	0.521	3.175
Depression	Absent/present	2.739	0.830	9.037
Anxiety	Absent/present	5.000	1.718	14.553
Stress	Absent/present	4.333	0.501	37.451
FSH	Deficiency/normal	3.000	1.180	7.627
LH	Deficiency/normal	4.500	1.902	10.647
Estradiol	Low/normal	0.364	0.156	0.850
TSH	High/normal	0.602	0.513	0.707
Testosterone	High/normal	0.583	0.493	0.691

While the odds ratio for BMI, depression, and stress occurring in secondary amenorrhea is greater than 1, the confidence interval includes 1. So, the association for these variables is insignificant. On the other hand, the odds ratio for anxiety, FSH, and LH levels is more than 1, and for estradiol, TSH, and testosterone levels is less than 1. In both cases, the interval does not include 1, which suggests that there is a significant association, although with variations.

## Discussion

In this case-control study, researchers emphasized BMI, stress, anxiety, depression, and serum levels of LH, FSH, prolactin, TSH, free T4, estradiol, and testosterone to find an association with secondary amenorrhea among the medical students of UMS.

Obesity is widely recognized as a factor that can lead to various gyneco-endocrine issues, such as hyperandrogenism, anovulation, polycystic ovary syndrome (PCOS), and fertility challenges. The resistin gene polymorphism has been linked to BMI among women with PCOS, indicating a potential connection between this genetic variation and adiposity in PCOS [34]. A randomized placebo-controlled study demonstrated that administering the insulin sensitizer rosiglitazone significantly reduced serum resistin levels among overweight women with PCOS [35]. However, in this study, researchers could not find any association between BMI and secondary amenorrhea, which corresponds to the findings of Silvestris et al. in 2018 and Aladashvili-Chikvaidze et al. in 2015 [34,36].

Functional hypothalamic amenorrhea (FHA) occurs in response to exaggerated metabolic, physical, or psychological stress such as severe dieting, heavy training, intense emotional events, or a combination of them with or without body weight loss. These stressors negatively affect GnRH release and the reproductive axis [37]. Reproductive functions and stress regulation systems are closely linked, and stress-related activation of the hypothalamic-pituitary-adrenal (HPA) axis leads to the inhibition of the hypothalamic-pituitary-ovarian (HPO) axis at multiple levels. Hypothalamic corticotropin-releasing hormone (CRH), the principal driving factor of the HPA axis during stress, is a potent inhibitor of GnRH secretion, which delays or suppresses LH secretion. Abnormal LH secretion can cause menstrual irregularities and amenorrhea [38,39]. Cortisol, the primary effector hormone of the HPA axis, is an important biological marker of stress and is released in a pulsatile manner [40]. Physical or mental stress activates an instant increase in CRH, and Valsamakis et al. reported that an elevated resting cortisol level during intense training was involved in the hypersecretion of CRH. Therefore, increased CRH causes menstrual disturbance through cortisol activation [41]. Acute stress impairs reproduction if it occurs at a critical time during the precise time course of endocrine events that induce estrus and ovulation. However, chronic stress impairs reproduction in general. Glucocorticoids exert a wide range of effects on metabolism, which are primarily catabolic to utilize every available energy resource against the challenge enforced by stressors, and chronic stress prolongs the adaptive shift towards a generalized catabolic state. Thus, sustained HPA hyperactivity can progressively lead to decreased lean (muscle and bone) body mass, increased visceral adiposity, and insulin resistance. Body weight and fat mass are independent regulators of the HPO axis activity, and disruption of homeostatic models by chronic life stressful events must be studied regarding PCOS endocrine dysregulation [42]. Participants of this study with secondary amenorrhea (case group) exhibited significantly higher (p>0.05) depression, anxiety, and stress levels than the control group. However, only the presence of anxiety increased the risk of secondary amenorrhea five times compared to the absence of it. In contrast, stress and depression could not predict the risk significantly, which corresponds to Sood et al. in 2012 and Clarvit in 1988 [15,26] but not to Harlow and Matanoski in 1991, Gordley et al. in 2000, Sherina et al. in 2004, Saipanish in 2003, Yamamoto in et al. 2009, and Tang et al. in 2020 [27-32]. In this study, most participants (90% or more) did not have stress or depression, but many participants (more than 30%) had moderate anxiety. According to the American Psychological Association, a subtle distinction exists between stress and anxiety. Both are emotional responses, but an external stimulus typically instigates stress [43]. Medical students experience stress most during examinations, and the participants only took part in this research during their off time; this factor could play a significant role in the fact that only a minority exhibited stress [44]. In contrast, anxiety is characterized by persistent and excessive concerns that persist without a specific stressor. Anxiety elicits a nearly identical array of symptoms as stress [43].

In this study, respondents with menstrual irregularity demonstrated higher E2, testosterone, and TSH levels and lower levels of LH than those with regular menstruation. The study also shows that if the FSH level decreases by 1 unit, there will be three times the risk of having secondary amenorrhea, whereas the risk is four times for LH (Table 7). Moreover, E2, testosterone, and TSH levels exhibited protective effects on secondary amenorrhea. The common denominator of FHA is an aberration of the pulsatile secretion of GnRH from the hypothalamus, ranging from undetectable pulses to variations in amplitude or frequency. These alterations result in the deregulation of FSH and LH levels, leading to decreased ovarian estradiol production and anovulation [45]. It has been reported that there is a link between FHA and PCOS. However, it is impossible to know whether this association between PCOS and FHA depends on some specific genetic trait or the involvement of some common hypothalamic mechanism. Some patients with FHA may have subtle increases in serum androgens during gonadotropin administration and, in addition, may have ovarian morphology like women with PCOS. In most studies, serum anti-Mullerian hormone (AMH) level, which is typical in FHA, has been reported to be increased. Increased AMH levels are an important diagnostic marker for altered ovarian morphology in women with PCOS [45].

This study had some limitations to consider when interpreting the results. First, most data on the participants' menstrual history, past medical history, and current weight gained or lost were collected through self-reported questionnaires, which were not confirmed by investigations and led to statistically significant correlation coefficients relatively weak. However, these correlations do not establish causation. An experimental study design is required to establish causation. Second, we could not measure the level of corticotropin-releasing hormone (CRH) and cortisol, which are principal driving factors of the HPA axis during stress. The level of these hormones has diurnal variations, and we could not fix the time to take blood samples because students could come to the clinic only when they were free from teaching schedules.

## Conclusions

The factors associated with secondary amenorrhea (PCOS and FHA) in medical students of this research were anxiety and hormonal influences, mainly deficiency of LH and FSH. Future studies should address the diurnal variation of the hormones and consider the participants' circumstances to get a proper effect of hormonal influence and stress.

## References

[1] Kafaei-Atrian M, Mohebbi-Dehnavi Z, Sayadi L, Asghari-Jafarabadi M, Karimian-Taheri Z, Afshar M (2019). The relationship between the duration of menstrual bleeding and obesity-related anthropometric indices in students. J Educ Health Promot.

[2] Roberts RE, Farahani L, Webber L, Jayasena C (2020). Current understanding of hypothalamic amenorrhoea. Ther Adv Endocrinol Metab.

[3] Zavatta G, Clarke BL (2021). Premenopausal osteoporosis: focus on the female athlete triad. Case Rep Womens Health.

[4] Coss D (2018). Regulation of reproduction via tight control of gonadotropin hormone levels. Mol Cell Endocrinol.

[5] Ramezani Tehrani F, Amiri M (2019). Polycystic ovary syndrome in adolescents: challenges in diagnosis and treatment. Int J Endocrinol Metab.

[6] Bae J, Park S, Kwon JW (2018). Factors associated with menstrual cycle irregularity and menopause. BMC Womens Health.

[7] Rosal MC, Ockene IS, Ockene JK, Barrett SV, Ma Y, Hebert JR (1997). A longitudinal study of students' depression at one medical school. Acad Med.

[8] Stewart SM, Lam TH, Betson CL, Wong CM, Wong AM (1999). A prospective analysis of stress and academic performance in the first two years of medical school. Med Educ.

[9] Singh G, Hankins M, Weinman JA (2004). Does medical school cause health anxiety and worry in medical students?. Med Educ.

[10] Wilkinson TJ, Gill DJ, Fitzjohn J, Palmer CL, Mulder RT (2006). The impact on students of adverse experiences during medical school. Med Teach.

[11] Styles WM (1993). Stress in undergraduate medical education: 'the mask of relaxed brilliance'. Br J Gen Pract.

[12] Abdulghani HM, AlKanhal AA, Mahmoud ES, Ponnamperuma GG, Alfaris EA (2011). Stress and its effects on medical students: a cross-sectional study at a college of medicine in Saudi Arabia. J Health Popul Nutr.

[13] Gazzaz ZJ, Baig M, Al Alhendi BS, Al Suliman MM, Al Alhendi AS, Al-Grad MS, Qurayshah MA (2018). Perceived stress, reasons for and sources of stress among medical students at Rabigh Medical College, King Abdulaziz University, Jeddah, Saudi Arabia. BMC Med Educ.

[14] Al Houri HN, Jomaa S, Arrouk DM, Nassif T, Al Ata Allah MJ, Al Houri AN, Latifeh Y (2023). The prevalence of stress among medical students in Syria and its association with social support: a cross-sectional study. BMC Psychiatry.

[15] Sood M, Devi A, Azlinawati Azlinawati (2012). Menses and stress related changes in female medical students. Procedia Soc Behav Sci.

[16] Hu FB (2008). Physical activity, sedentary behaviors, and obesity. Obesity Epidemiology.

[17] Mehmood Y, Al-Swailmi FK, Al-Enazi SA (2016). Frequency of obesity and comorbidities in medical students. Pak J Med Sci.

[18] Gopalakrishnan S, Ganeshkumar P, Prakash MV, Christopher Christopher, Amalraj V (2012). Prevalence of overweight/obesity among the medical students, Malaysia. Med J Malaysia.

[19] Boo NY, Chia GJ, Wong LC, Chew RM, Chong W, Loo RC (2010). The prevalence of obesity among clinical students in a Malaysian medical school. Singapore Med J.

[20] Ekpenyong CE, Davis KJ, Akpan UP, Daniel NE (2011). Academic stress and menstrual disorders among female undergraduates in Uyo, South Eastern Nigeria - the need for health education. Niger J Physiol Sci.

[21] Zhou M, Wege N, Gu H, Shang L, Li J, Siegrist J (2010). Work and family stress is associated with menstrual disorders but not with fibrocystic changes: cross-sectional findings in Chinese working women. J Occup Health.

[22] Rafique N, Al-Sheikh MH (2018). Prevalence of menstrual problems and their association with psychological stress in young female students studying health sciences. Saudi Med J.

[23] Karout N, Hawai SM, Altuwaijri S (2012). Prevalence and pattern of menstrual disorders among Lebanese nursing students. East Mediterr Health J.

[24] Nisar N, Zehra N, Haider G, Munir AA, Sohoo NA (2008). Frequency, intensity and impact of premenstrual syndrome in medical students. J Coll Physicians Surg Pak.

[25] Issa BA, Yussuf AD, Olatinwo AW, Ighodalo M (2010). Premenstrual dysphoric disorder among medical students of a Nigerian university. Ann Afr Med.

[26] Clarvit SR (1988). Stress and menstrual dysfunction in medical students. Psychosomatics.

[27] Harlow SD, Matanoski GM (1991). The association between weight, physical activity, and stress and variation in the length of the menstrual cycle. Am J Epidemiol.

[28] Gordley LB, Lemasters G, Simpson SR, Yiin JH (2000). Menstrual disorders and occupational, stress, and racial factors among military personnel. J Occup Environ Med.

[29] Sherina MS, Rampal L, Kaneson N (2004). Psychological stress among undergraduate medical students. Med J Malaysia.

[30] Saipanish R (2003). Stress among medical students in a Thai medical school. Med Teach.

[31] Yamamoto K, Okazaki A, Sakamoto Y, Funatsu M (2009). The relationship between premenstrual symptoms, menstrual pain, irregular menstrual cycles, and psychosocial stress among Japanese college students. J Physiol Anthropol.

[32] Tang Y, Chen Y, Feng H, Zhu C, Tong M, Chen Q (2020). Is body mass index associated with irregular menstruation: a questionnaire study?. BMC Womens Health.

[33] Dean AG, Arner TG, Sunki GG (2023). Epi Info 2000: a database, and statistics program for public health professionals for use on Windows 95, 98, and NT computers. https://stacks.cdc.gov/view/cdc/23207.

[34] Silvestris E, de Pergola G, Rosania R, Loverro G (2018). Obesity as disruptor of the female fertility. Reprod Biol Endocrinol.

[35] Spicer LJ, Schreiber NB, Lagaly DV, Aad PY, Douthit LB, Grado-Ahuir JA (2011). Effect of resistin on granulosa and theca cell function in cattle. Anim Reprod Sci.

[36] Aladashvili-Chikvaidze N, Kristesashvili J, Gegechkori M (2015). Types of reproductive disorders in underweight and overweight young females and correlations of respective hormonal changes with BMI. Iran J Reprod Med.

[37] Podfigurna A, Maciejewska-Jeske M, Meczekalski B, Genazzani AD (2020). Kisspeptin and LH pulsatility in patients with functional hypothalamic amenorrhea. Endocrine.

[38] Iwasa T, Matsuzaki T, Yano K, Mayila Y, Irahara M (2018). The roles of kisspeptin and gonadotropin inhibitory hormone in stress-induced reproductive disorders. Endocr J.

[39] Miyamoto M, Hanatani Y, Shibuya K (2021). Relationship among nutritional intake, anxiety, and menstrual irregularity in elite rowers. Nutrients.

[40] Gifford RM, O'Leary TJ, Double RL (2019). Positive adaptation of HPA axis function in women during 44 weeks of infantry-based military training. Psychoneuroendocrinology.

[41] Valsamakis G, Chrousos G, Mastorakos G (2019). Stress, female reproduction and pregnancy. Psychoneuroendocrinology.

[42] 43] American Psychological Association (2023). What's the difference between stress and anxiety?. https://www.apa.org/topics/stress/anxiety-difference.

[43] Bergmann C, Muth T, Loerbroks A (2019). Medical students' perceptions of stress due to academic studies and its interrelationships with other domains of life: a qualitative study. Med Educ Online.

[44] Kyriakidis M, Caetano L, Anastasiadou N, Karasu T, Lashen H (2016). Functional hypothalamic amenorrhoea: leptin treatment, dietary intervention and counselling as alternatives to traditional practice - systematic review. Eur J Obstet Gynecol Reprod Biol.

[45] Carmina E, Fruzzetti F, Lobo RA (2016). Increased anti-Mullerian hormone levels and ovarian size in a subgroup of women with functional hypothalamic amenorrhea: further identification of the link between polycystic ovary syndrome and functional hypothalamic amenorrhea. Am J Obstet Gynecol.

